# How standardised are antibiotic regimens in otologic surgery?

**DOI:** 10.1186/s40463-023-00669-y

**Published:** 2023-11-09

**Authors:** Justin T. Lui, Valerie Dahm, Christoph Arnoldner, Philip W. Lam, Trung N. Le, Joseph M. Chen, Vincent Y. Lin

**Affiliations:** 1 Section of Otolaryngology–Head and Neck Surgery, Department of Surgery, Cumming School of Medicine, University of Calgary, Calgary, Canada; 2 Department of Otolaryngology–Head and Neck Surgery, Medical University of Vienna, Währinger Gürtel 18-20, 1090 Vienna, Austria; 3 Division of Infectious Diseases, Department of Medicine, Sunnybrook Health Sciences Centre, University of Toronto, Toronto, Canada; 4 Deparment of Otolaryngology–Head and Neck Surgery, Sunnybrook Health Sciences Centre, University of Toronto, Toronto, Canada

**Keywords:** Antibiotics, Antimicrobial resistance, Otology, Cochlear implant, Middle ear, Quality assurance

## Abstract

**Background:**

Within otologic surgery, a paucity of well-controlled studies assessing the use of systemic antibiotic to reduce surgical site infections exists. Moreover, discrepancies in wound classification of procedures challenge consensus in antimicrobial prescribing patterns. We sought to compare surgeons from two different health systems to examine how surgeons’ prescribing habits compared to practice guidelines for numerous otologic procedures.

**Methods:**

An online questionnaire was distributed to 33 Canadian and 32 Austrian surgeons who regularly perform otologic surgery. Current systemic antibiotic prescribing habits for cochlear implantation, cholesteatoma surgery, stapes surgery, and tympanoplasty ± ossiculoplasty were collected.

**Results:**

Eighteen of 33 (54.5%) Canadian surgeons provided responses, while 18 of 32 (56.3%) of Austrian surgeons answered. Clear consistency with clinical practice guidelines exists for pre-operative antibiotics use in cochlear implant surgery and infected cholesteatoma surgery. However, for stapes surgery and tympanoplasty ± ossiculoplasty, consensus is lacking for both pre- and post-operative antibiotic prescribing habits. Notable differences between the two countries include post-operative antibiotics for cochlear implant surgery (Austria: 36.4%, Canada: 71.4%) and uninfected cholesteatoma surgery (Austria: 33.3%, Canada: 77.8%). Across all procedures, both induction and post-operative antibiotic administration was not significantly associated with surgeon seniority when stratified by five-year increments.

**Conclusion:**

The lack of consensus among each country’s otologic surgeons underscores the uncertainty in wound classification and thus, adherence to clinical practice guidelines.

**Graphical Abstract:**

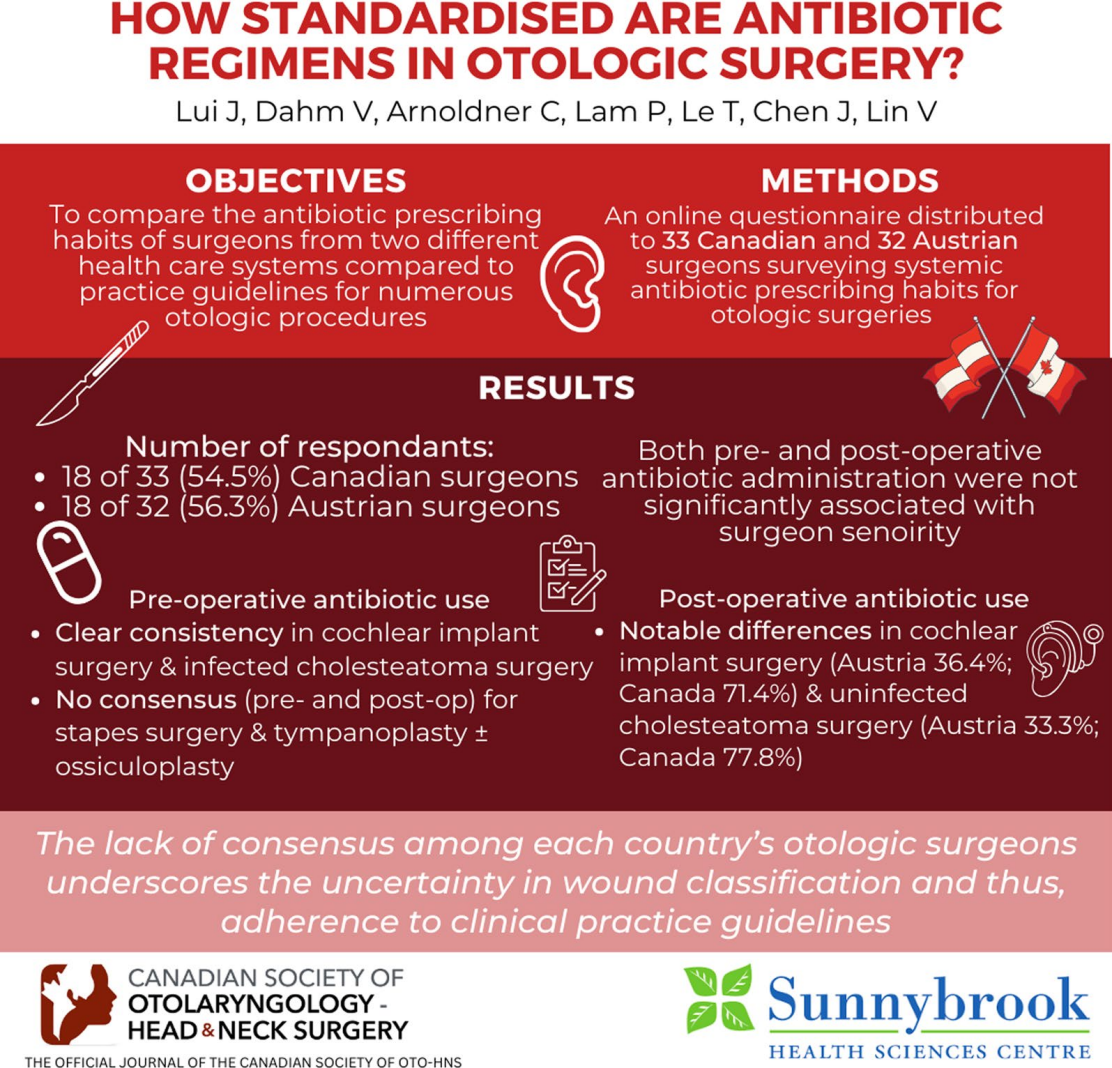

**Supplementary Information:**

The online version contains supplementary material available at 10.1186/s40463-023-00669-y.

## Background

The fear of post-surgical infections causing prolonged medical and surgical intervention may prompt the indiscriminate use of antibiotics described as a “protective umbrella” [1]. Medicolegal ramifications may influence surgeon administration or prescription of systemic perioperative antibiotics. For example, routine cochlear implant (CI) surgery lacks sufficient evidence to warrant routine prophylactic antibiotics. However, given the risks of meningitis and costly replacement of infected devices, patients often receive systemic antibiotics as prophylaxis [2].

To address the emerging global health crisis of antimicrobial resistance, the World Health Organization (WHO) has established “options for action”. Within this report, hospital antimicrobial stewardship is listed as a key pillar in addressing this twenty-first century crisis. Expedited by antibiotics misuse in both humans and animals, antibiotic resistance has contributed to increased healthcare expenditures and increasingly difficult to treat bacterial infections. Additionally, adverse effects from antibiotics may arise from misuse in the form of *C. difficile infections* and drug-associated toxicities such as acute kidney injury [3].

The Centers for Disease Control and Prevention (CDC) has established a wound classification that stratifies surgical site infections (SSIs) risk [4]. Based off a prospective cohort study of 62,939 wounds, four classes were established: clean, clean-contaminated, contaminated, and dirty. This stratification was linked to a SSI rate of < 2%, < 10%, 20%, and 40% [4]. As a result, antibiotic prophylaxis is advocated for the latter three classes.

Within the domain of otologic surgery, a paucity of well-controlled studies assessing the use of systemic antibiotic to reduce surgical site infections exists [5]. A recent Cochrane review assessing antibiotic prophylaxis in clean and clean-contaminated otologic surgery included only four eligible randomized controlled trials [6]. Given the lack of clear evidence driven recommendations, significant disparities between surgeon prescribing patterns exist despite published guidelines [5, 7].

Otologic surgical site infections (SSI’s) range from 1 to 4%, which is in keeping with the 0.5 to 3% of SSI rates in all US surgeries [8–10]. Post-stapedotomy or tympanoplasty (dry perforations) SSI’s were 3.9%, while patients undergoing tympanoplasty or tympanomastoidectomy for chronic ear disease was estimated to be 5.3% [8, 9]. A recent meta-analysis on cochlear implants and wound infections reported a rate of 1.36% of infections in the adult population and 1.45% in pediatric patients, which comprised over 21,838 implants [11].

We set out to assess the antibiotic prescribing patterns between two vastly different health systems being Canada and Austria for multiple otologic procedures including cochlear implantation, cholesteatoma surgery, tympanoplasty, ossiculoplasty, and stapes surgery. Key differences do exist between both systems including the outpatient focus in Canada, which contrasts a more inpatient focus in Austria. As a result, different antibiotics are more readily available to Austrian surgeons which can be given iv postoperatively. Further differences include reimbursement: in Canada many physicians are paid case by case while in Austria all surgeons are salaried. Most otologic surgeries in both countries are carried out by otologists with in-depth training in the field. A further important difference especially for cochlear implantation surgery is the distance patients need to travel to reach the hospital. Austria is a small country with many hospitals and short travel distances, Canada on the other hand is a significantly bigger country with a very centralized health care system. Not being able or being able to assess your patients regularly might also influence antibiotic prescribing patterns. The comparison of these two countries may provide a framework for other countries to assess their standing in antibiotic prescribing patterns. Moreover, it may identify similarities and differences between and within each health system.

## Methods

An online questionnaire was distributed to 33 Canadian and 32 Austrian surgeons who regularly perform otologic surgery. Surgeons were identified through internal databases of registrants to both national societies of Otolaryngology–Head & Neck Surgery. This 30-question survey remained open from November 2020 to January 2021 (Additional file 1: Supplemental Digital Content). Surgeon demographics including age, gender, and years of independent experience were captured. Additionally, current systemic antibiotic prescribing habits for cochlear implantation, cholesteatoma surgery, stapes surgery, and tympanoplasty ± ossiculoplasty were collected. The survey platform, Survey Monkey (San Mateo, USA), was employed for survey distribution, response collection, and preliminary analysis. This investigation was exempt from human ethics review by both research boards given its role as a quality assurance and improvement initiative.

The first iteration of the survey was scrutinized by four otologists and one infectious disease physician to ensure face validity. Double-barreled, loaded, or confusing questions were removed. The finalized version was translated into German by two Austrian Otologists. Bivariate analysis was performed by a statistician employing a Fisher’s exact test. Incentives were not offered, and completion was purely on a voluntary basis.

## Results

**Fig. 1:**
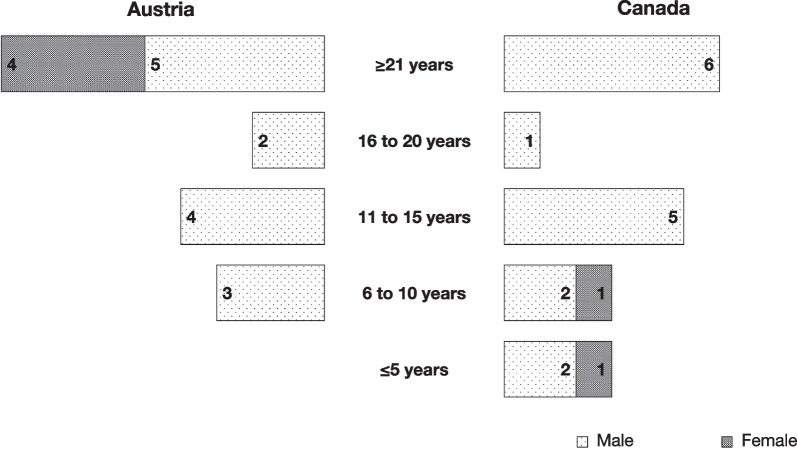
Demographics of respondents separated by country and experience

A total of 36 responses were included out of a possible 65 with 2 survey results omitted given incomplete responses. Eighteen of 33 (54.5%) Canadian surgeons provided responses, while 18 of 32 (56.3%) of Austrian surgeons responded (Fig. 1). Male respondents outweighed females 27 to 6. Practice distribution was nearly equivalent when subdividing respondents by greater or less than 15 years of experience (48.5% vs. 51.5%). Table 1 demonstrates perioperative systemic antimicrobial use separated by procedure and country.

**Table 1:** Pre- and post-operative systemic antibiotics stratified by procedure and country

Rate of antibiotic use	Operative phase	Austria		Canada	
Cochlear Implant surgery	Induction	10/11	90.9%	9/9	100%
	Post-operative	4/11	36.4%	7/9	77.8%
Dry cholesteatoma surgery	Induction	13/18	72.2%	13/18	72.2%
	Post-operative	7/18	38.9%	3/18	16.7%
Infected cholesteatoma surgery	Induction	13/18	100.0%	16/18	88.9%
	Post-operative	17/18	94.4%	14/18	77.8%
Stapedotomy	Induction	12/17	70.6%	6/16	37.5%
	Post-operative	2/17	11.8%	3/16	18.8%
Tympanoplasty	Induction	10/18	55.6%	9/18	50.0%
	Post-operative	5/18	27.8%	4/18	22.2%
Tympanoplasty and ossiculoplasty	Induction	11/18	61.1%	9/18	50.0%
	Post-operative	7/18	38.9%	4/18	22.2%

### CI surgery

Of the 20 surgeons routinely performing cochlear implant surgery, 95.0% use pre-incision induction intravenous antibiotics. Of the Canadian surgeons, cefazolin was used in most cases (88.9%), while Austrian surgeons were divided between amoxicillin-clavulanate (30.0%), cefazolin (30.0%), and cefuroxime (40.0%). There was a significant association between country and type of pre-operative antibiotic (*p* < 0.01).

The routine use of post-operative antibiotics is greater in Canada (77.8% vs. 36.4%, *p* < 0.01). The post-operative antibiotics used by Canadian surgeons included amoxicillin-clavulanate (20.0%), cefazolin (20.0%), cephalexin (40.0%), and minocycline (20.0%). Among Austrian CI surgeons, the majority prescribed intravenous amoxicillin-clavulanate, which transitioned to oral form upon discharge.

### Cholesteatoma surgery

Endaural and post-auricular approaches were most common for attic cholesteatomas. There were no endoscopic surgeons amongst the Austrian respondents. For extensive cholesteatomas, 97% of all surgeons preferred a post-auricular approach.

Outlined in Table 1, both Austrian and Canadian surgeons’ use of induction antibiotics increased when faced with an infected cholesteatoma (72.2–100.0% and 72.2–88.9%, respectively). Similarly, to other procedures, cefazolin was the antibiotic of choice for Canadian respondents contrasting Austrian respondents varied choices. The antibiotic preference, however, did change for Austrians with actively draining cholesteatomas warranting the increased use of piperacillin-tazobactam from 7.7 to 38.9%. For all respondents, the use of post-operative antibiotics increased given the presence of a draining, infected cholesteatoma. The use of post-operative antibiotics increased from 38.9 to 94.4% for Austrian surgeons and 16.7–77.8% for Canadian surgeons.

### Tympanoplasty ± ossiculoplasty

Although equally favoured as the transcanal approach in Canada, the endaural approach was most employed by the Austrian surgeons with nearly half purporting its use followed by a post-auricular approach (38.9%). Respondents in both countries were nearly equally split with induction antibiotics for tympanoplasty. When factoring in ossiculoplasty, one respondent switched to prescribing induction antibiotics. Similarly, to stapes surgery, there was significant variation of antibiotics among Austrian respondents with five different antibiotics selected. Cefazolin was the most common pre-incisional antibiotic used in Canada, while Amoxicillin-Clavulanate was preferred in Austria.

In Austria, the rate of post-operative systemic antibiotic administration for tympanoplasty was 27.8%, which increased to 38.9% with ossiculoplasty. Contrastingly, the minority of Canadian surgeons (22.2%) prescribe post-operative antibiotics for tympanoplasty ± ossiculoplasty.

### Stapes surgery

Surgical approaches were different between countries with an endaural approach favored in Austria (64.7%) and transcanal favored in Canada (62.5%). Similar differences existed between countries as Austrian surgeons were more likely to prescribe pre-incisional antibiotics (70.6% vs. 37.5%) with cefuroxime (40.0%) being the most prescribed followed by amoxicillin-clavulanate (30.0%) and cefazolin (30.0%). The 37.5% of Canadian surgeons utilizing pre-incisional antibiotics all opted for cefazolin. Post-operatively, 78.8% of all surgeons did not prescribe post-operative antibiotics. The preferred post-operative antibiotic was Amoxicillin-Clavulanate (75%). Both induction and post-operative antibiotic selections were not linked with any country.

### Experience and approaches

Canadian otologists who answered the survey had the following years of experience in independent practice: ≤ 5 years (16.7%), 6 to 10 years (16.7%), 11 to 15 years (27.8%), 16 to 20 years (5.6%) and ≥ 21 years (33.3%). Austrian otologists had the following years of experience in independent practice: ≤ 5 years (0%), 6 to 10 years (16.7%), 11 to 15 years (22.2%), 16 to 20 years (11%) and ≥ 21 years (50%).

Across all procedures, both induction and post-operative antibiotic administration was not significantly associated with surgeon seniority when stratified by five-year increments.

## Discussion

A major challenge in guiding antimicrobial prescriptions for otologic procedures is the lack of widely accepted contamination classification schemes [5, 12, 13]. Even when using the Centre for Disease Control and Prevention’s classification of clean, clean-contaminated, contaminated, and dirty-infected, consensus of where otologic procedures fit is lacking. This challenge exists within other surgical domains evidenced by low inter-rate reliability [14].

Meta-analyzed data in the 2009 Cochrane update grouped tympanoplasty, stapes surgery, and mastoidectomy as clean otologic procedures in the absence of purulent ear discharge [6]. Routine antibiotics would not be indicated except one pre-incisional dose for CI surgery [5, 7]. An argument exists, however, that considers most otologic surgery as clean-contaminated since the middle ear is contiguous with the pharynx via the Eustachian tube and is covered by respiratory epithelium [12]. For clean-contaminated surgery, only a single course of induction antibiotics would be warranted [5]. This discrepancy would explain the lack of consensus within both countries otologists for the use of induction antibiotics in stapes surgery, tympanoplasty, and tympanoplasty with ossiculoplasty. While most Austrian surgeons prescribed induction antibiotics for these procedures (70.6%, 55.6%, 61.1%), most Canadian surgeons either did not prescribe induction antibiotics or remained evenly divided for these procedures (37.5%, 50.0%, 50.0%). Despite the lack of indications for post-operative antibiotics for both clean or clean-contaminated classification, a minority of respondents utilized post-operative antibiotics [5, 7].

Cholesteatoma surgery may be differentiated by the presence of infection, which would change its clean-contaminated status to dirty-infected [5, 6]. A contrasting categorization considers all cholesteatoma surgery as dirty-infected [13]. Some consensus exists between both countries as the majority of surgeons considered infected cholesteatoma as dirty-infected procedures requiring induction and post-operative antibiotics. Dry cholesteatoma lacking purulent debris, however, was a source of differentiation. Irrespective of the contamination classification, induction antibiotics are suggested since dry cholesteatoma surgery is considered at least clean-contaminated. In cases considered contaminated or dirty-infected, a pre-operative dose of antibiotics is linked with a lower post-operative surgical site infection rate [13]. Post-operatively, Canadians were more likely to see dry cholesteatoma surgery as dirty-infected (77.8%) versus Austrians (33.3%) given the use of post-operative antibiotics (Fig. 1). The confusion with classification may be a source of discrepancy in prescribing behaviours of surgeons.

Despite notable differences in prescribing habits between Austrian and Canadian otologists, statistically significant differences were lacking. Only one other published investigation of otologic antibiotic prescribing habits was identified, which evaluated 81 Australian and New Zealand surgeons [15]. Notably, respondents were less likely to use pre-operative antibiotics for CI surgery (62.1%) in comparison to our investigational data. Use of pre- and postoperative antibiotics for stapes surgery was 41.0% and 43.0%, respectively. Comparable prescribing rates for pre- and post-procedural antibiotics were seen for tympanoplasty and ossiculoplasty at 47.2% and 31.0%, respectively. Although the infection status of cholesteatoma surgery was not specified, the Australian/New Zealand surgeons tended to use less induction antibiotics (44.4%) the Austrian and Canadian surgeons in our investigation.

**Fig. 2:**
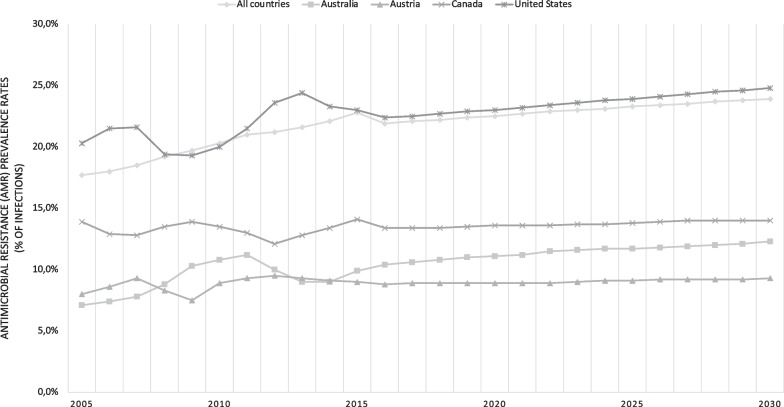
Projected antimicrobial resistance prevalence rates from 2005 to 2030 as per the Organization for Economic Co-operation and Development (OECD)

Judicious use of antibiotics is a key pillar in mitigating the health burden antimicrobial resistance (AMR) [16]. Viewed as one of the greatest global health threats to humanity, AMR is challenged by the lack of novel antimicrobials to address pathogens such as carbapenem-resistant gram negatives or third generation cephalosporin-resistant *K. pneumoniae* [16]. Based on the Organisation for Economic Co-operation and Development’s projections of 52 countries, nearly one quarter of all infections will be resistant to antimicrobial treatment for eight antibiotic-bacterium combinations (Fig. 2) [16]. Even in high-income countries with lower AMR prevalence such as Australia, Austria, and Canada, increased morbidity and mortality are projected unless effective policies are in place to stem the tide [16]. Namely, effectiveness of antibiotic prophylaxis for common procedures while making post-operative infections more difficult to treat [16].

Given the AMR’s extrinsic threat to global health, surgeons’ assistance in antimicrobial stewardship is imperative. From running well-controlled trials that strengthen antibiotic prophylaxis clinical guidelines to participating in hospital and community infection control programs, numerous opportunities exist for surgeons. Moreover, initiatives such as the “Choosing Wisely,” campaign helps champion antibiotic stewardship by producing informed, evidence-based guidelines to align prescribing patterns while improving patient literacy with antimicrobials.

To support antimicrobial stewardship efforts, the authors believe clearly defining the otologic procedural contamination classification is imperative in combating AMR. Clean surgeries encompass middle ear and canal-wall up mastoid procedures such as tympanoplasty, ossiculoplasty, stapedotomy, and cochlear implantation unless surgical site sterilization cannot be performed. Perioperative antibiotics are not warranted for clean surgeries except for one prophylactic dose for CI surgery [5]. Dry cholesteatoma surgery may be considered contaminated, warranting intraoperative antibiotics followed by a short course of postoperative antibiotics [5, 13]. Procedures involving culture proven infection or the presence of purulence would warrant a dirty classification, which would involve both pre- and post-operative antibiotics.

Several limitations of this investigation exist. Despite the lack of interviewer bias, web-based surveys are subject to a non-response bias. In this investigation, nearly 44.6% of eligible surgeons did not respond to the survey despite reminder notifications. Additionally, the survey did not undergo rigorous principal components analysis nor assessed for internal consistency to prove internal validity. Finally, since our focus was on evaluating the use of systemic antibiotics, we did not capture the use of topical antibiotics.

## Conclusion

This investigation highlights significant discrepancy that exists within a relatively niche subspecialty regarding antibiotic use. Despite published guidelines, adherence is lacking especially in light of confusion that exists regarding the SSI risk classification of otologic surgeries. Clarity of classification is imperative to achieve uniformity and reduce AMR. Continued efforts in producing well-designed clinical controlled trials comparing SSI outcomes between different antimicrobial protocols will help shape and strengthen guidelines. Moreover, education programs and national specialty societies should aim at collaborating to produce well-adhered guidelines. Continued efforts to mitigate the effects of AMR are imperative and may have tremendous impacts on future generations.

### Supplementary Information

**Additional file 1.** 30 question survey.

## Data Availability

Available upon request to the corresponding author.

## Abbreviations

AMR: Antimicrobial resistance
CDC: Centers for Disease Control and Prevention
CI: Cochlear implant
OECD: Organization for Economic Co-operation and Development
SSI: Surgical site infections
WHO: World Health Organization
