# Supplementary material for: How standardised are antibiotic regimens in otologic surgery?
Source: J Otolaryngol Head Neck Surg. 2023 Nov 9;52:73. doi: 10.1186/s40463-023-00669-y (PMC10634121; doi:10.1186/s40463-023-00669-y)
Supplement: Supplementary file 1 — Additional file 1. 30 question survey. [file 40463_2023_669_MOESM1_ESM.pdf]

Dear Colleague,

Thank you for taking the time to complete this brief questionnaire regarding your practice. The responses will remain entirely anonymous.

We understand that some of the questions may not accurately capture all of your experiences, however, we do ask that you try and answer questions to the best of your abilities (i.e. represents ≥75% of your practice).

If you have any questions, concerns, or remarks, please feel free to contact me at vincent.lin@sunnybrook.ca.

Warm regards,

Vincent Lin MD, FRCSC

* 1. In what setting do you work?

- [ ] Academic
- [ ] Community

* 2. What is your gender?

- [ ] Female
- [ ] Male
- [ ] Prefer not to say

* 3. How many years of independent practice have you completed?

- [ ] ≤5 years
- [ ] 6 to 10 years
- [ ] 11 to 15 years
- [ ] 15 to 20 years
- [ ] >21 years

* 4. How many of your ear surgeries do you perform under conscious sedation (not including tympanostomy tube insertion)

0% Percent 100%

5. I routinely perform cochlear Implant surgery:

6. I routinely give IV antibiotics during **INDUCTION** for cochlear implant surgery:

	Yes	No
Answer		

If antibiotic not listed:

7. I routinely give systemic antibiotics **POST-OPERATIVELY** for cochlear implant surgery:

	Yes	Duration	No
Answer			

If antibiotic not listed:

8. I routinely prescribe the following analgesic therapy **AFTER** cochlear implant surgery (Please select **ALL** that apply):

- [ ] Acetaminophen
- [ ] Celecoxib
- [ ] Diclofenac
- [ ] Ibuprofen
- [ ] Naproxen
- [ ] Codeine/Tylenol 3 & 4
- [ ] Hydromorphone/Vicodin
- [ ] Morphine
- [ ] Oxycodone/Percocet
- [ ] Tramadol/Tramacet

9. I routinely inject local anesthetic for **POST-OPERATIVE** analgesia at the end of cochlear implantation:

	Yes	No
Answer		

* 10. I am confident that my prescribed analgesics provides adequate pain control.

-10 (strongly disagree) 0 (neutral) 10 (strongly agree)

11. I routinely perform stapedotomy:

12. My **PREFERRED** (≥75% of cases) approach for stapedotomy is:

- [ ] Endaural
- [ ] Transcanal
- [ ] Endoscopic
- [ ] Postauricular

13. I routinely give IV antibiotics during **INDUCTION** for stapedotomy.

	Yes	No
Answer		

If antibiotic not listed

14. I routinely give systemic antibiotics **POST-OPERATIVELY** for stapedotomy.

	Yes	Duration	No
Answer			

If antibiotic not listed:

15. I routinely prescribe the following analgesics **POST-OPERATIVELY** for stapedotomy (Please select **AL**L that apply):

- [ ] Acetaminophen
- [ ] Celecoxib
- [ ] Diclofenac
- [ ] Ibuprofen
- [ ] Naproxen
- [ ] Codeine/Tylenol 3 & 4
- [ ] Hydromorphone/Vicodin
- [ ] Morphine
- [ ] Oxycodone/Percocet
- [ ] Tramadol/Tramacet

* 16. I am confident that my prescribed analgesics provides adequate pain control.

-10 (strongly disagree) 0 (neutral) 10 (strongly agree)

17. I routinely perform tympanoplasty:

18. My **PREFERRED** (≥75% of cases) approach for tympanoplasty is:

19. I routinely prescribe IV antibiotics during **INDUCTION** for tympanoplasty:

	TM perforation	w/ Ossiculoplasty
Answer		

If antibiotic not listed:

20. I routinely prescribe systemic antibiotics **POST-OPERATIVELY** for tympanoplasty?

	TM perforation	w/ ossiculoplasty	Duration
Answer			

If antibiotic not listed:

21. I routinely inject local anesthetic for **POST-OPERATIVE** analgesia at the end of tympanoplasty:

	Yes	No
Answer		

22. I routinely prescribe the following analgesics **POST-OPERATIVELY** for tympanoplasty (Please select **ALL** that apply):

- Acetaminophen
- Celecoxib
- Diclofenac
- Ibuprofen
- Naproxen
- Codeine/Tylenol 3 & 4
- Hydromorphone/Vicodin
- Morphine
- Oxycodone/Percocet
- Tramadol/Tramacet

* 23. I am confident that my prescribed analgesics provides adequate pain control.

-10 (strongly disagree) 0 (neutral) 10 (strongly agree)

24. I routinely perform cholesteatoma surgery

25. My **PREFERRED** (≥75% of cases) approach for **LIMITED** cholesteatoma (e.g. attic) surgery is:

- ☐ Endoscopic
- ☐ Retro/Postauricular
- ☐ Endaural

26. My **PREFERRED** (≥75% of cases) approach for **EXTENSIVE** cholesteatoma (e.g. involving mastoid) surgery is:

- ☐ Endoscopic
- ☐ Retro/postauricular
- ☐ Endaural

27. I routinely give IV antibiotics during **INDUCTION** for cholesteatoma surgery:

	Dry cholesteatoma	Infected cholesteatoma
Answer		

If antibiotic not listed:

28. I routinely give systemic antibiotics **POST-OPERATIVELY** for cholesteatoma surgery:

	Dry cholesteatoma	Infected cholesteatoma	Duration
Answer			

If antibiotic not listed:

29. Do you have any comments?

Liebe Kolleginnen und Kollegen!

Vielen herzlichen Dank, dass Sie sich die Zeit für diesen kurzen Fragebogen nehmen.

Nicht jede Frage und Antwort kann jedes Detail ihrer Praxis widerspiegeln. Wir bitten Sie die best mögliche Antwort, welche ca. 75% ihrer täglichen Praxis abdeckt, auszuwählen. Die Antworten bleiben natürlich anonym.

Sollte es Fragen oder Anliegen geben, wenden Sie sich gerne an valerie.dahm@meduniwien.ac.at.

Ihr,

Christoph Arnoldner

* 1. In welchem Umfeld arbeiten Sie?

- [ ] Hauptsächlich öffentliches Krankenhaus
- [ ] Hauptsächlich Ordination und Privatspital
- [ ] 50% öffentliches Krankenhaus, 50% Privatspital

* 2. Sind sie..

- [ ] Weiblich
- [ ] Männlich
- [ ] möchte ich nicht bekannt geben

* 3. Seit wie vielen Jahren sind Sie HNO-Fachärztin/Facharzt?

- [ ] ≤5 Jahre
- [ ] 6 bis 10 Jahre
- [ ] 11 bis 15 Jahre
- [ ] 15 bis 20 Jahre
- [ ] >21 Jahre

* 4. Wieviele der von Ihnen durchgeführten Ohroperationen führen Sie in lokaler Betäubung durch? (Parazentese und Paukendrainage ausgenommen)

0% Prozent 100%

* 5. Führen Sie regelmässig Cochlea Implantationen durch?

6. Verabreichen Sie routinemäßig eine intravenöse **PERIOPERATIVE antimikrobielle Prophylaxe** (kurz vor OP Beginn) bei Cochlea Implantationen?

	Ja	Nein
Antwort		

7. Verabreichen Sie meist eine **INTRAVENÖSE POSTOPERATIVE** antimikrobielle Prophylaxe nach Cochlea Implantation (z.B.: während des weiteren Krankenhaus Aufenthaltes)?

	Ja	Nein	Dauer
Antwort			

8. Verschreiben Sie Ihren Patienten routinemäßig eine **POSTOPERATIVE** antimikrobielle Prophylaxe **PER OS** nach Cochlea Implantationen?

	Ja	Nein	Dauer
Antwort			

9. Welche Medikamente verschreiben Sie meist zur **postoperative Schmerztherapie** nach Cochlea Implantation (bitte **ALLE** auswählen die zutreffen)?

- ☐ Paracetamol
- ☐ Celecoxib
- ☐ Diclofenac
- ☐ Ibuprofen
- ☐ Naproxen
- ☐ Mefenaminsäure (Parkemed, Mefenam,...)
- ☐ Metamizol (Novalgin)
- ☐ Morphin
- ☐ Oxycodon
- ☐ Tramadol/Tramal
- ☐ Hydal
- ☐ Codein
- ☐ Anderes

10. Injizieren Sie **AM ENDE** der Cochlea Implantation ein Lokalanästhetikum zur Analgesie?

	Ja	Nein
Antwort		

* 11. Wie sicher sind Sie, dass Ihre Patienten mit dieser von Ihnen verschriebenen Analgetika Therapie ausreichend schmerzfrei sind?

0 (nicht sicher) — 10 (sehr sicher)

* 12. Führen Sie regelmässig Stapesplastiken durch?

13. Ich bevorzuge (>75%) folgenden **ZUGANG** für Stapesplastiken:

- [ ] Endaural
- [ ] "Transcanal"
- [ ] Endoskopisch
- [ ] Retro/postaurikulär

14. Verabreichen Sie routinemäßig eine **INTRAVENÖSE PERIOPERATIVE antimikrobielle Prophylaxe** (kurz vor OP Beginn) bei Stapesplastiken?

	Ja	Nein
Antwort		

15. Verabreichen Sie meist eine **INTRAVENÖSE POSTOPERATIVE** antimikrobielle Prophylaxe nach Stapesplastiken (z.B.: während des weiteren Krankenhaus Aufenthaltes)?

	Ja	Nein	Dauer
Antwort			

16. Verschreiben Sie Ihren Patienten routinemäßig eine **POSTOPERATIVE** antimikrobielle Prophylaxe **PER OS** nach Stapesplastiken?

	Ja	Nein	Dauer
Antwort			

17. Injizieren Sie **AM ENDE** der Stapesplastik ein Lokalanästhetikum zur Analgesie?

	Ja	Nein
Antwort		

18. Welche Medikamente verschreiben Sie hauptsächlich zur postoperative **Schmerztherapie** nach einer Stapesplastik (bitte **ALLE** auswählen die zutreffen)?

- [ ] Paracetamol
- [ ] Celecoxib
- [ ] Diclofenac
- [ ] Ibuprofen
- [ ] Naproxen
- [ ] Mefenaminsäure (Parkemed, Mefenam,..)
- [ ] Metamizol (Novalgin)
- [ ] Morphin
- [ ] Oxycodon
- [ ] Tramadol/Tramal
- [ ] Hydal
- [ ] Codein
- [ ] Anderes

* 19. Wie sicher sind Sie, dass Ihre Patienten mit dieser von Ihnen verschriebenen Analgetika Therapie ausreichend schmerzfrei sind?

0 10

* 20. Führen Sie regelmässig Tympanoplastiken durch?

21. Welchen **ZUGANG** bevorzugen (>75%) Sie für Tympanoplastiken?

22. Verabreichen Sie ein Antibiotikum zur **INTRAVENÖSE PERIOPERATIVE antimikrobielle Prophylaxe** (kurz vor OP Beginn) bei Tympanoplastiken?

	Reine Trommelfellperforation	Mit Ossikuloplastik
Antwort		

23. Verschreiben Sie Ihren Patienten meist eine **POSTOPERATIVE INTRAVENÖSE** antimikrobielle Prophylaxe nach folgenden Operationen? (zum Beispiel für die Dauer des Krankenhaus-Aufenthaltes)

	Reine Trommelfellperforation	Mit Ossikuloplastik	Dauer
Antwort			

24. Verschreiben Sie Ihren Patienten meist eine **POSTOPERATIVE** antimikrobielle Prophylaxe **PER OS** nach folgenden Operationen?

	Reine Trommelfellperforation	Mit Ossikuloplastik	Dauer
Antwort			

25. Injizieren Sie **am Ende** der Tympanoplastik ein Lokalanästhetikum zur Analgesie?

	Ja	Nein
Antwort		

26. Welche Medikamente verschreiben Sie meist zur **postoperative Schmerztherapie** nach Tympanoplastik (bitte **ALLE** auswählen die zutreffen)?

- ☐ Paracetamol
- ☐ Celecoxib
- ☐ Diclofenac
- ☐ Ibuprofen
- ☐ Naproxen
- ☐ Mefenaminsäure (Parkemed, Mefenam,..)
- ☐ Metamizol (Novalgin)
- ☐ Morphin
- ☐ Oxycodon
- ☐ Tramadol/Tramal
- ☐ Hydal
- ☐ Codein
- ☐ Anderes

* 27. Wie sicher sind Sie, dass Ihre Patienten mit dieser von Ihnen verschriebenen Analgetika Therapie ausreichend schmerzfrei sind?

0 10

* 28. Führen Sie regelmässig Cholesteatom-Operationen durch?

29. Ich bevorzuge (>75%) folgenden **ZUGANG** für **limitierte Cholesteatome** (z.B. Attik)

30. Ich bevorzuge (>75%) folgenden **ZUGANG** für **ausgeprägte/große Cholesteatome** (z.B. Mastoidbeteiligung)

31. Verabreichen Sie meist eine **INTRAVENÖSE PERIOPERATIVE antimikrobielle Prophylaxe** (kurz vor OP Beginn) vor Cholesteatom Eingriffen?

	Cholesteatom (trockenes Ohr)	Superinfiziertes Cholesteatoma
Antwort		

32. Verabreichen Sie meist eine **INTRAVENÖSE POSTOPERATIVE** antimikrobielle Prophylaxe nach Cholesteatom Eingriff?

	Cholesteatom (trockenes Ohr)	Superinfiziertes Cholesteatom	Dauer
Antwort			

33. Verabreichen Sie meist eine **POSTOPERATIVE** antimikrobielle Prophylaxe **PER OS** nach Cholesteatom Eingriff?

	Cholesteatom (trockenes Ohr)	Superinfiziertes Cholesteatom	Dauer
Antwort			

34. Vielen Dank für Ihre Zeit. Möchten Sie uns gerne etwas mitteilen?
